# How can health technology assessment support our response to public health emergencies?

**DOI:** 10.1186/s12961-022-00925-z

**Published:** 2022-11-04

**Authors:** Aparna Ananthakrishnan, Alia Cynthia Gonzales Luz, Sarin KC, Leslie Ong, Cecilia Oh, Wanrudee Isaranuwatchai, Saudamini Vishwanath Dabak, Yot Teerawattananon, Hugo C. Turner

**Affiliations:** 1 Health Intervention and Technology Assessment Program (HITAP), Ministry of Public Health, Nonthaburi, Thailand; 2 Access and Delivery Partnership, United Nations Development Programme (UNDP), Bangkok, Thailand; 3 Institute of Health Policy, Management and Evaluation, University of Toronto, Toronto, ON Canada; 4 Saw Swee Hock School of Public Health, National University of Singapore, Singapore, Singapore; 5 MRC Centre for Global Infectious Disease Analysis, School of Public Health, Imperial College London, London, United Kingdom

**Keywords:** Health technology assessment, Public health emergencies, COVID-19, Health policy, Economic evaluation, Evidence-informed policy-making, Universal health coverage, Pandemic preparedness

## Abstract

Public health emergencies (PHEs), such as the COVID-19 crisis, are threats to global health and public order. We recommend that countries bolster their PHE responses by investing in health technology assessment (HTA), defined as a systematic process of gathering pertinent information on and evaluating health technologies from a medical, economic, social and ethical standpoint. We present examples of how HTA organizations in low- and middle-income countries have adapted to supporting PHE-related decisions during COVID-19 and describe the ways HTA can help the response to a PHE. In turn, we advocate for HTA capacity to be further developed globally and for increased institutional acceptance of these methods as a building block for preparedness and response to future PHEs. Finally, the long-term potential of HTA in strengthening health systems and embedding confidence and transparency into scientific policy should be recognized.

## Introduction

Public health emergencies (PHEs) are threats to global health, healthcare systems and public order that can arise from several causes, including the outbreaks of contagious, life-threatening diseases, natural disasters, among others [1]. In January 2020, the COVID-19 outbreak in Wuhan, China, was declared a public health emergency of international concern (PHEIC) by the World Health Organization (WHO). COVID-19 has since been recognized as a pandemic, with its crippling impact transcending national boundaries and paralyzing global freedoms and economies. The current crisis has shown that a PHE of this scale is rarely limited to health outcomes alone and quickly escalates into an economic, ethical and social catastrophe [2]. Since the start of the pandemic, decision-makers have had to make urgent recommendations about the use of pharmaceutical and non-pharmaceutical interventions (NPIs) (such as national and regional border closures, social distancing measures including commercial and business restrictions, personal protection strategies such as masks and hand hygiene) as well as, more recently, COVID-19 vaccines. Each of these decisions have required a careful balance, such as between implementing strict public health measures while protecting the economy and livelihoods, between public safety and individual freedoms, and in a broader sense, between short-term and long-term regional and global development.

For policy responses to be effective and accrue higher benefits than costs, they must be tailored to local settings and consider all potential intended and unintended consequences on health and other sectors [3]. The timely use of the best available evidence, deliberated in a participatory and transparent manner, therefore becomes a prerequisite for successful policy implementation. An evidence-informed response helps in (1) improving the understanding of potential implications of available policy options on different population groups, (2) highlighting gaps in current knowledge and indicating areas for further research or technical capacity strengthening and (3) enhancing credibility, legitimacy and accountability of decisions to promote public acceptance of, confidence in [4] and adherence to the resulting policies [5, 6]. On the contrary, when scientific evidence is isolated from decision-making, the consequences are severe as has been witnessed in serious outbreaks across a few countries [7].

In this article, we recommend that countries bolster their PHE responses by adopting and investing in health technology assessment (HTA). HTA is “a multidisciplinary process that uses explicit methods to determine the value of a health technology at different points in its lifecycle” [8, 9]. The difficulty in prioritizing scarce resources has contributed to many of the challenges faced by countries throughout the pandemic. Some prominent examples include the efficient allocation of the limited supply of personal protective equipment, critical care equipment, vaccine stocks, drugs and treatments between patient groups, hospitals, or at the macro level, between sectors such as population health and the economy, and most conspicuously, between countries and regions. The core principles of HTA are designed precisely to address such challenges. HTA presents evidence highlighting the costs and benefits of all available options and serves as a tool for decision-makers by enabling evidence-informed decision-making in a transparent manner [10]. Furthermore, HTA processes engage a diverse group of stakeholders throughout the process and facilitate knowledge dissemination channels which can be critical in addressing misinformation around COVID-19 and the interventions used to contain outbreaks. The COVID-19 crisis has highlighted the essential role of HTA in tackling PHEs by effectively promoting “an equitable, efficient, and high-quality health system” [9, 11]. The use of HTA to inform decision-making during the pandemic has demonstrated the urgent emphasis on socio-ethical considerations beyond the immediate health impacts, and offered insights and lessons on methodological improvements that will be needed for an improved response to future crises.

In this paper, we focus on select examples from HTA organizations in low- and middle-income countries (LMICs) in adapting their existing expertise, networks and resources to support PHE-related decisions. These are based on a three-part webinar series titled “Knowledge Exchange in the time of COVID-19: Using Evidence to Address Health Care Challenges in Low- and Middle-Income Countries” organized by the Access and Delivery Partnership (ADP) and the Health Intervention and Technology Assessment Program (HITAP) [12]. The knowledge gained from the webinar series as well as literature showcasing the value of HTAs in informing policy decisions [13–15] inspires our call for strengthening HTA processes and their institutionalization to address ongoing and future prioritization issues.

### How can HTA contribute to a better PHE response?

Prior to the pandemic, agencies conducting HTA supported their governments on several national-level policies, a majority of which focused on chronic health conditions. However, since 2020, HTA has contributed to addressing COVID-19 issues in several ways. This led to an interest in and consideration of ways in which the HTA approach can be applied to support the response to emergencies. The following are some examples:

**Generating and gathering evidence to support timely and informed decisions** Since the beginning of the pandemic, countries have struggled to make timely and evidence-informed decisions about the introduction of COVID-19 pharmaceuticals, vaccines and other preventative or curative interventions. Many governments have created national teams or task forces to evaluate and synthesize information to support these responses. Given their technical capacity and national focus, HTA agencies in many countries have been assisting the evidence needs of these task forces on conducting disease modelling, performing rapid reviews and interviews with key national and international experts, as well as other types of healthcare research, as required. In the Philippines, the HTA Unit under the Department of Health supported the assessment of COVID-19 technologies for review by the Inter-Agency Task Force for Emerging Infectious Diseases to inform the development of COVID-19-relevant policies [16]. The assessment generated critical evidence on the use of rapid antigen kits for COVID-19 diagnosis, provision of drugs such as favipiravir and Cycloferon, and of the AstraZeneca vaccine [17]. Similar studies in Thailand helped prioritize the limited tranche of COVID-19 vaccine stocks between different population groups in the country using available data to support the implementation of the vaccination programme [18]. These experiences show the value of strengthening processes/mechanisms/policies to conduct scientifically rigorous research and empowering national research agencies which can support policy and programmatic priorities, respond promptly to evidence generation needs and contribute to clear and informed decision-making.

**Appraising the value of decisions** Governments aim to maximize the health outcomes of their investments and collating such evidence even before interventions are launched in the market can be critical in PHEs. One approach that is being piloted across countries is early HTA, defined as “all methods used to inform industry and other stakeholders about the potential value of new medical products in development, including methods to quantify and manage uncertainty” [19, 20] which has been useful in exploring the optimal mix of COVID-19 vaccines and NPIs. HTA agencies in Kenya, Thailand and Singapore conducted an assessment of hypothetical COVID-19 vaccines based on existing vaccine product profiles to understand at which levels and types of vaccine efficacy (e.g. preventing transmission, severe disease or disease contraction), and their combination with NPIs, would best address their respective countries’ healthcare needs [20]. This type of research is useful not only to generate contextually relevant evidence but also to discuss issues of vaccine development and procurement with industry and supranational initiatives such as the COVID-19 Vaccines Global Access (COVAX) [21]. Countries such as Lao People's Democratic Republic, Ghana and the Philippines are also interested in using HTA to conduct economic evaluations of COVID-19 vaccines to inform prioritization of target populations and the adaptation of vaccine strategies to address emerging disease variants, among others. Studies on the cost-effectiveness of different NPIs have also been a promising area of research; for example, in India, hand hygiene was found to be cost-effective, while public avoidance of surgical masks and respirator use “could save resources" [22].

The substantial economic impact of COVID-19 (with estimates ranging from US$ 77 billion to US$ 2.7 trillion globally [23]) is important to factor into policy decisions as well. Many NPIs such as social distancing, national lockdowns and border closures have significant financial implications. Given that the poor and vulnerable groups are often the hardest hit during PHEs (for example, around 22 million people in sub-Saharan Africa are estimated to be pushed into extreme poverty, with the cost likely falling disproportionately on women, who globally lost around US$ 800 billion in income in 2020 [23, 24]), the inclusion of ethical and social considerations within the HTA process ensures that these populations can be accounted for.

**Identifying knowledge gaps and priority research areas** Urgent national responses to the COVID-19 pandemic have meant that much of the available health resources have been channelled away from key priority areas, exacerbating critical gaps within the healthcare system. This has compelled governments and healthcare decision-makers to focus on priority areas and implement efficient, streamlined efforts to adequately deliver all existing healthcare demands, including COVID-19. Here, HTA research has been able to support governments in understanding these challenges, as well as informing decisions concerning resource allocation [25]. In India, for example, Prinja et al. estimated a 2.5–3.8% increase in deaths from cervical cancer due to delays in diagnosis and treatment [26]. This type of research can identify clinical areas which require closer monitoring and evaluation to ensure that health outcomes can be stabilized and even improved. On the other hand, it also highlights areas of lower priority or low-value care, for example, “medical services that provide little to no clinical benefit or may cause harm to patients such as antibiotic use for a likely uncomplicated viral infection or imaging for nonspecific low-back pain” [27]. The pandemic could be a catalyst for health professionals and policy-makers to re-prioritize resource allocation or re-evaluate the values that govern decision-making processes for resource allocation [27–30]. Oncology practices in Canada, the United Kingdom, and the United States, for example, now use a “priority-based approach to cancer care”, whereby treatments with clear evidence of improved survival or quality of life are prioritized. This approach has led to a significant reduction in the use of treatments which exhibit small clinical benefits but a high probability of hospitalization and severe side effects. Such efforts aim to balance clinical needs of the patient with requisite treatment while respecting resource limitations and social distancing without compromising clinical benefit [29, 31].

**Stronger governance and policy response** Established HTA processes can be adapted in the context of a PHE, contributing to stronger governance and policy response. In this context, the HTA principles of transparency and accountability of both researchers and policy-makers are particularly important. For example, based on HTA processes, guidance regarding the use of modelling for informing policy responses to COVID-19 policy was developed [32]. This included standards on how the outputs of the modelling are reported and guidance on facilitating collaboration between modellers/researchers and decision-makers [32], to promote the use of modelling evidence to inform decision-making. This highlights the importance of established HTA processes in facilitating the effective use of evidence and knowledge to inform a comprehensive policy response to PHEs.

**Legitimizing government decisions and instilling public trust** The process used within HTA is designed to be transparent and inclusive. This can be vital for health policies to be trusted, understood and accepted by stakeholders, including the general public. In Thailand, for instance, HITAP developed a prioritization protocol in the event of shortages of critical care and intensive care units resulting from a sudden increase in the number of severe COVID-19 patients. With the extensive involvement of relevant stakeholders (including religious leaders, clinicians and ethicists) in the decision-making process, the overarching need to prioritize “maximizing total benefits for the society” was identified and agreed upon as the primary ethical principle guiding the decision-making for critical care resources [33]. These types of consensus-building across prioritization and decision-making processes are vital and must be transparently communicated to broader stakeholder groups such as the public through open lines of communication. Another recent example is a regional study from Asia which analysed vaccine certification uptake through public and institutional surveys, allowing citizen participation and support for government policy-making on the issue from these results [34]. This builds confidence in and bolsters the government’s efforts to address challenging healthcare needs during emergencies. An inclusive and participatory approach can be key to policy acceptance, given the sensitive nature of the issue and the potential risk of public distrust. Unverified and incorrect information can spread quickly and widely over the course of PHEs, impeding public health responses and leading to further disease spread; this phenomenon of too much or harmful misinformation during a PHE is referred to as an “infodemic” by WHO [35, 36]. For example, fake news, misinformation and conspiracy theories have skyrocketed since the beginning of the COVID-19 pandemic, fuelled by the ubiquity of social media [12]. The transparent and evidence-based approaches used within the HTA process can help reduce the risk and spread of such misinformation [37, 38].

The preceding examples collectively illustrate the multiple ways in which HTA, as an institutional approach and as a scientific discipline, can help ensure a country is well placed to address PHEs. This also highlights that HTA is not simply about economic evaluations but a wider framework for the evaluation and use of evidence to inform decision-making.

## Conclusions

The COVID-19 pandemic has re-emphasized the value of data to maximize health outcomes. The novelty of the severe acute respiratory syndrome coronavirus 2 (SARS-CoV-2) virus has meant that many aspects associated with it are constantly evolving, including critical information on viral mutations, efficacy and side effects of treatments and vaccines [39]. All these pieces of evidence have significant implications on social and economic policies and practices. Scientific approaches with the capability of including such wide-ranging evidence are therefore essential to inform decision-making in times of crisis. In this paper, we suggest HTA is one such approach as it operates within a framework that recognizes many vital parameters and interactions of evidence-informed decision-making. In support, we reference several examples to show that well-established HTA processes and techniques have been able to support immediate, actionable structures for pandemic response. In addressing our current crisis, HTA processes have facilitated the flow of research skills across disciplines and departments, breaking long-standing silos within healthcare research. They have also shown promise in offering adaptive frameworks to suit contextual requirements, especially useful for resource-constrained settings [40].

That said, HTA is by no means a panacea. One common criticism is the complexity of HTA and the lack of clear guidance for the evaluation of various interventions; Bluher et al. (2019) outline these issues using the example of medical devices in the European Union. For novel crises such as COVID-19, this might require urgent standards to be established and updated with changing circumstances [41]. As an analytic approach relying on diverse, yet specific, knowledge types and contributing stakeholders, HTA processes also require strong knowledge generation and epistemological systems to be in place, which are especially challenging in under-resourced contexts [42]. Importantly, HTA mechanisms are ensconced within the wider ecosystem of economic and political interests which can strongly influence scientific decision-making. For instance, differential power relations between stakeholder groups, particularly in less transparent HTA systems, could result in limited inclusion of the views of marginal groups across the entire process; an important example is patient involvement within HTA [43, 44]. Institutionalizing HTA processes within this macrocosm, such as by establishing HTA research agencies, requires strong political and institutional buy-in, potentially with a legislative foundation that embeds the process of HTA into policy-making [45]. Such strong leadership is also vital in ensuring that all findings and recommendations from HTA processes are appropriately interpreted, accepted, communicated and implemented, after which they are consistently monitored and fed back into the process for improved decisions. For instance, good national COVID-19 performance has not necessarily followed conventional assumptions wherein higher-income countries outperform those less affluent [46]; this could be considered a function of societal values in priority-setting impacted by the influences of interest groups and underlying societal structures [47]. The secure mediatory presence of an independent, interdisciplinary, technical agency to holistically inform policy actions and receive support from institutional or legal mandates has shown to be a useful arrangement, especially in times of emergencies. As we expand our understanding of the public health discipline to recognize other PHEs such as gun violence, antimicrobial resistance, air pollution and climate change events as realities, there is growing interest in the ability of HTA to comprehensively address these questions.

In immediate progress, strong research collaborations during the pandemic across disciplines have been able to recognize and learn from shortfalls of current modelling methods and HTA systems [48]. HTA agencies and interest groups are taking steps to prepare adaptable HTA methodologies that respond to urgent needs despite the paucity of data and the rapid changes in information that are characteristic of pandemics or other health emergency situations. For example, the "Best-practice guidance for the health technology assessment of diagnostics and treatments for COVID-19" shares recommendations on the use of "living" clinical reviews and other HTA models that analyse newly published data as they are produced and reduce duplication efforts [49]. Outlining similar pragmatic approaches for HTA agencies, the document also suggests simpler methods such as cost-consequence analyses to assess value-for-money considerations without using frequently unavailable utility values. These adaptations are demonstrative of customization within the discipline and also deeply valuable for LMICs that face such challenges even during nonemergency situations. A key example of technical learning in the field includes the recognition that economic evaluations for COVID-19 policy responses, and arguably other PHEs, are inappropriate in limiting their analyses to the health systems perspective alone and warrant a society-wide calculation, meaning that non-health costs/benefits of NPIs and other such measures are just as critical to analyse [50]. In their paper, Painter et al. further elaborate on the important potential of economic evaluations on COVID-19 vaccines and the technical considerations in conducting these studies, building on case experiences from Singapore and Thailand. Another example relates to the weights given to different cost inputs within calculations based on immediate disease-related conditions; in situations where intensive care unit (ICU) beds are fully occupied, they are costed with greater opportunity costs than other factors such as labour and materials, given their fundamental life-saving ability for patients [51]. These developments showcase promise for the approach in serving as a ready tool during crises.

As the pandemic continues and new health security challenges emerge, healthcare practitioners and policy-makers are required to regularly re-prioritize their efforts. This pattern has been mirrored by HTA agencies as well, where important HTA research on non-pandemic health priorities and routine services including immunizations such as rotavirus and pneumococcal vaccines have been paused. This paper argues that there is an imminent need to build technical expertise and capacity for HTA globally, as the concepts and processes of HTA hold immense value in prioritizing and addressing policy-relevant questions. The unique aspect of HTA in aggregating inputs from diverse stakeholders, generating and appraising such relevant evidence and then communicating the final results to decision-makers, including the public, is urgently required during PHEs. From COVID-19 and other similar crises, it is clear that these practices are fully aligned with recommended actions for governments in response to any or every PHE. The benefits of applying HTA concepts using existing infrastructure (even without dedicated HTA bodies) for decision-making are especially useful in LMICs which face a chronic shortage of healthcare resources and incur higher opportunity costs from poor decisions. Over the course of the pandemic, regional and global networks for learning and sharing have emerged as important avenues of translational research and evidence use. For instance, collaborative networks and platforms (such as HTAsiaLink, Guide to Health Economics Analysis and Research, and ADP/HITAP knowledge exchange initiatives on HTA that include the webinar series on COVID 19 among others [12, 52]), foster participation and learning for policy-makers, researchers and other stakeholders involved in HTA processes. They have offered upskilling opportunities such as trainings and workshops as well as lessons sharing events on important issues such as policy advocacy, guideline development and other contextual challenges. We emphasize that a commitment for greater financial resources towards HTA, while reinforcing political will and institutional acceptance of these methods are building blocks for preparedness and response to future PHEs. More importantly, we must recognize the long-term potential of HTA in strengthening health systems and embedding confidence and transparency into scientific policy decision-making. These impacts are far-reaching as an important component of universal health coverage (UHC) and, broadly, the Sustainable Development Goals [53].

## Data Availability

Not applicable.

## Abbreviations

ADP: Access and Delivery Partnership
COVAX: COVID-19 Vaccines Global Access
HITAP: Health Intervention and Technology Assessment Program
HTA: Health technology assessment
LMICs: Low- and middle-income countries
NPIs: Non-pharmaceutical interventions
PHEs: Public health emergencies
PHEIC: Public health emergency of international concern
UHC: Universal health coverage
WHO: World Health Organization
